# Bibliometric analysis of global research on physical activity and sedentary behavior in the context of cancer

**DOI:** 10.3389/fonc.2023.1095852

**Published:** 2023-01-26

**Authors:** Jialin Gu, Miao Hu, Yonglin Chen, Jialin Yu, Yi Ji, Guoli Wei, Jiege Huo

**Affiliations:** ^1^ Department of Oncology, Affiliated Hospital of Integrated Traditional Chinese and Western Medicine, Nanjing University of Chinese Medicine, Nanjing, Jiangsu, China; ^2^ The Third Clinical Medical College, Nanjing University of Chinese Medicine, Nanjing, Jiangsu, China; ^3^ Key Laboratory of Acupuncture and Medicine Research of Ministry of Education, Nanjing University of Chinese Medicine, Nanjing, Jiangsu, China; ^4^ Department of Oncology, Jiangsu Province Academy of Traditional Chinese Medicine, Nanjing, Jiangsu, China

**Keywords:** sedentary behavior, physical activity, cancer, bibliometrics analysis, global research

## Abstract

**Objective:**

Numerous studies focusing on sedentary behavior (SB) and physical activity (PA) in the context of cancer have been reported in recent years. We analyzed and visualized studies on SB and PA in patients with cancer over the last 20 years using scientometric methods, to provide insights on gaps and deficiencies in the literature, and to inform future research guidelines.

**Methods:**

All relevant studies in the field from 2001 to October 2022 were reviewed using bibliometric tools, including VOSviewer, Bibliometric online analysis platform, and biblioshiny, to determine the most influential countries, institutions, journals, and authors. We explored current research hotpots and potential research trends, based on keyword clustering and dynamic changes. Our research focuses on PA, SB, and cancer across the entire cancer continuum, from primary prevention to treatment to cancer survivorship.

**Results:**

Scientometric analysis identified 4,382 relevant manuscripts on SB and PA in the context of cancer, with a 10-fold increase in articles over the past 20 years. The United States, Canada, and Australia were the most influential countries. The journal, *Supportive Care in Cancer*, had the highest number of publications, while *Clinical Oncology* had the highest H-index. K.S. Courneya was the most influential author in this field, with the highest number of publications, total citations, and H-index. Keyword analysis revealed that current research is focused on PA and SB in patients with breast cancer, quality of life, and aerobic exercise. Future frontiers include cancer prehabilitation programs and cardiorespiratory fitness, and remote intervention and social support.

**Conclusion:**

By using bibliometrics, we conducted a comprehensive review of SB and PA in cancer-related studies. The current research focused on exercise and sedentariness in breast cancer patients and the role of PA in improving quality of life in survivorship. Emerging research foci were generally around cancer prehabilitation programs and remote intervention issues for PA. In addition, some publication deficits are noted: studies of PA and SB in less common cancers; the recommended doses and intensities of exercise for cancer; the timing of interventions for prehabilitation and the establishment of individualized exercise protocols. These deficiencies align with the needs for future research topics.

## 1 Introduction

With 19.3 million new cases and 10 million deaths in 2020, cancer is a leading cause of premature death and a significant barrier to increasing life expectancy for the global population (1, 2), and results in high social medical expenses and personal financial burden (3, 4). To date, cancer remains a major public problem that threatens the health of the population. Increased average life expectancy and the prevalence of unhealthy lifestyles, such as sedentary behavior (SB), high intake of tobacco and alcohol, and non-Mediterranean Western dietary patterns, characterized by high sugar and calorie content, have further increased cancer risk (5). The widespread prevalence of these risk factors among young adults is also a potential cause of the progressively earlier age of cancer onset (6). Advances in early screening and treatment technologies have benefited a subset of cancer survivors who have longer survival times; hence, the need to ensure the quality of life (QoL) for cancer survivors has become an essential consideration for clinicians (7). Moreover, reduced levels of physical activity (PA) due to a sedentary lifestyle are a non-negligible cause of cancer (8, 9).

In recent years, researchers have begun to focus on the significance of modifiable behavioral factors in cancer development and progression, particularly the potential associations among SB, PA, and cancer. Numerous epidemiological studies have found that appropriate PA reduces the risk and improves the prognosis of several cancers, while SB has the exact opposite effect (10, 11). Nevertheless, SB and low PA levels are becoming increasingly common in modern lifestyles, both in the healthy population and among cancer survivors. In 2018, the American College of Sports Medicine conducted a roundtable on the correlations between PA, SB, and cancer, which reported on the negative significance of SB in relation to cancer occurrence and prognosis, while providing preliminary evidence to support the positive role of PA in preventing multiple cancers and increasing the life expectancy of cancer survivors (12). Exercise oncology has received increasing attention in the field of cancer treatment and supportive care (13, 14). However, the effective translation of exercise oncology into clinical practice remains a challenge. Given the potential value of these modifiable behavioral factors in primary cancer prevention and supportive cancer care, numerous publications have emerged; however, the accumulation of publications has also caused difficulties for researchers in identifying current research hotspots and future frontier issues. Therefore, new patterns of literature search are urgently needed.

Bibliometrics is a method involving qualitative and quantitative analysis of publications in specific fields, which uses a combination of mathematics and statistics to identify basic features, knowledge structures, current hotspots, and research frontiers (15, 16). Bibliometrics can be used to identify the most influential countries, authors, institutions, journals, and publications, synthesizing and visualizing key information from different disciplines (17–20). There have been several scientometric studies on exercise and cancer, including on the molecular mechanisms underlying the effects of exercise on cancer (21) and rehabilitation exercise in patients with cancer (22); however, no studies providing a more comprehensive and systematic scientometric analysis of this field have been conducted. We therefore hypothesize that bibliometrics can provide a comprehensive analysis and assessment of research hotspots and future frontiers in this field. To test our hypothesis above, we conducted a knowledge mapping study of publications related to SB and PA in the context of cancer to summarise (i) details of the top authors, countries, institutions, and journals. (ii) insights on areas of robust publication and apparent deficits. (iii) emerging research trends and future frontier in this field. In particular, our research focuses on PA, and SB across the entire cancer continuum, as these modifiable factors play an essential role in cancer research from primary prevention to treatment to survivorship. The results will enable investigators to better align their research inquiries and help funding agencies understand topical and emerging research areas.

## 2 Materials and methods

### 2.1 Bibliometric search strategy

**Figure 1:**
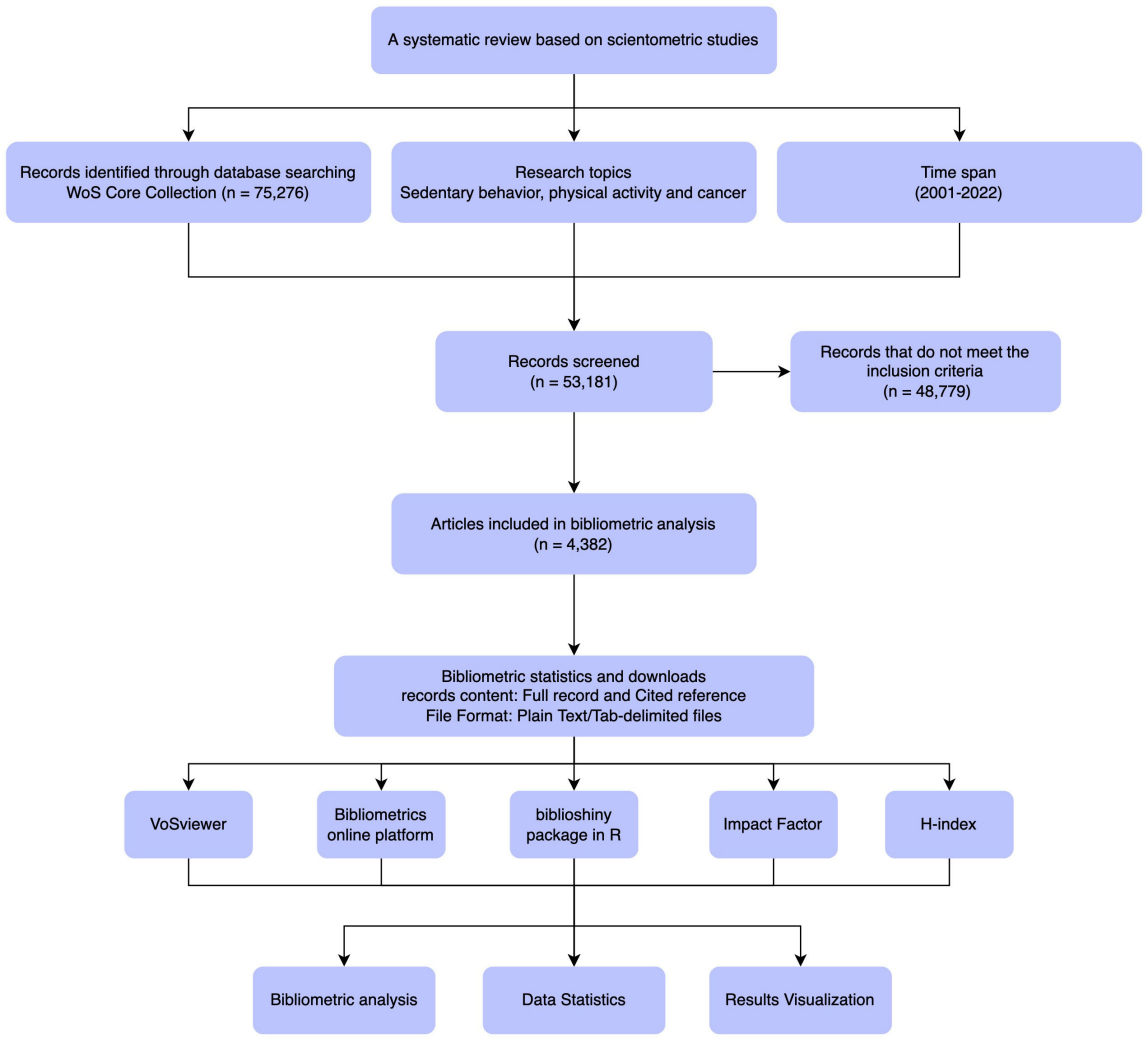
Flow chart of study identification and selection.

Our primary question concerned the 20-year research trends in PA, SB, and cancer. In our initial review, we found that publications related to PA and SB in the context of cancer are relatively scarce before 2001. Therefore, eligible studies published from January 2001 to October 2022 were retrieved from the Web of Science Core Collection (WoSCC, Clarivate Analytics). The WoSCC database provides a comprehensive data source for scientometric analysis and is currently the most used database for this type of research (17, 23). The specific search strategy is presented in **Supplementary Table 1**. Only manuscripts published in English were considered, and publication types selected were articles and reviews; conference abstracts, editorial material, letters to the editor, and comments were excluded. Titles and abstracts were screened by two independent reviewers. A flow chart for study identification and selection is shown in **Figure 1**.

### 2.2 Data statistics and visualization

Key data for publications that met the criteria, including research title, author, research institution, country/region, journal, publication year, number of citations, 2021 impact factor (IF), and references, were downloaded from WoSCC. Microsoft Excel 2020, OriginPro 2021, VOSviewer (24), the R language package, biblioshiny (25), and the Bibliometric online analysis platform (https://bibliometric.com/) were used for data analysis and visualization. In addition, the H-index (numbers of both published and cited papers ≥ h) was used to identify high-quality authors and manuscripts (26); the H-index is a key bibliometric indicator used to assess the overall research performance of researchers, journals, institutions, or countries (27, 28).

## 3 Results

### 3.1 Annual Overview of Publications

**Figure 2:**
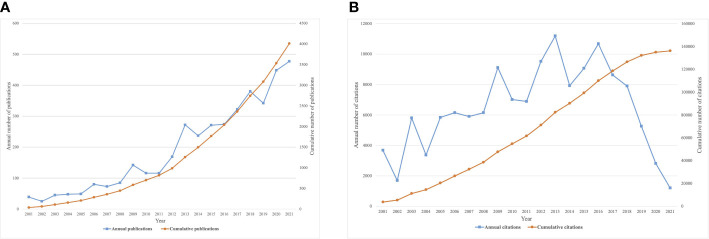
Number of publications and citations of SB, PA, and cancer. **(A)** Annual and cumulative number of publications; **(B)** Annual and cumulative number of citations.

A total of 4,382 publications were identified as meeting the criteria after a review of titles and abstracts (81% of original studies and 19% of reviews). Annual numbers of publications on SB and PA in cancer have increased significantly over the last two decades, from 39 in 2001 to 477 in 2021 (**Figure 2A**). Based on current trends, annual publications are expected to reach 500 in 2025. In addition, the number of citations has gradually increased and peaked in 2013 and 2016 (**Figure 2B**).

### 3.2 Distribution of countries/regions

**Figure 3:**
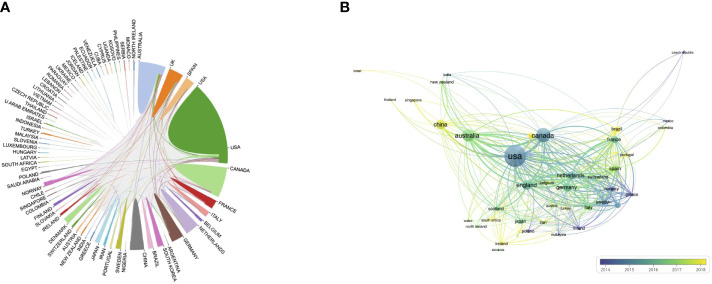
Distribution of countries associated with SB, PA, and cancer. **(A)** International collaboration between countries/regions **(B)** Temporal trend of country/region collaboration.

In total, authors from 77 countries and territories contributed to research advances in the field of PA and SB in the context of cancer, with the largest number of publications coming from the United States (39.1%), followed by Canada (16.4%), Australia (12.5%), the United Kingdom (7.8%), China (7.8%), and Germany (6.6%). We also analyzed the cooperation relationships between countries (**Figure 3A**). The results showed closer collaboration between the United States, Canada, and the United Kingdom, and less collaboration between China and other countries. We also found that the United States and Canada have been conducting research in this field for a longer period of time than China and Australia (**Figure 3B**). In terms of citations, publications from the United States had the highest citation frequency (n = 65,133), followed by Canada (n = 30,986), Australia (n = 17,058), and the United Kingdom (n = 14,118).

### 3.3 Journal distribution

**Figure 4:**
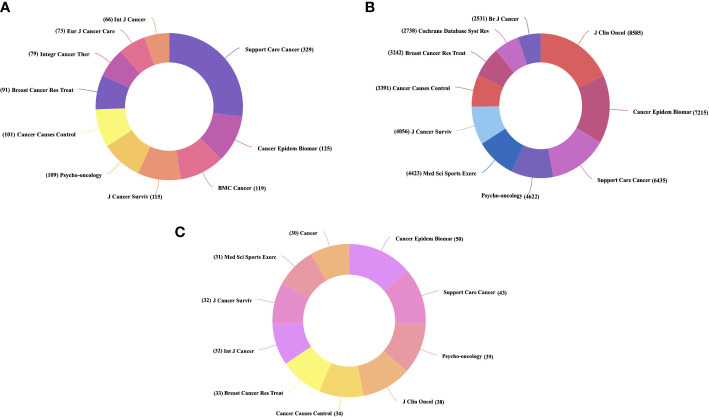
Distribution of Journals associated with SB, PA, and cancer. **(A)** Top 10 journals with the highest number of publications **(B)** Top 10 journals with the highest number of citations **(C)** Top 10 journals with the highest H-index.

A total of 756 journals published research on PA and SB in cancer during our study period. The top 10 major journals published a total of 1207 publications. Among them, Supportive Care in Cancer had the highest number of publications (n = 329), followed by Cancer Epidemiology Biomarkers & Prevention (n = 125), and BMC Cancer (n = 119) (**Figure 4A**). In terms of citations, the Journal of Clinical Oncology, which is among the most influential journals in the field of oncology, ranked first, with 46 publications receiving 8585 citations, followed by Cancer Epidemiology Biomarkers & Prevention (7215 citations), Supportive Care in Cancer (7215 citations), and Psycho-Oncology (6435 citations) (**Figure 4B**). In addition, Cancer Epidemiology Biomarkers & Prevention had the highest H-index, indicating the higher quality of manuscripts published in this journal (**Figure 4C**).

### 3.4 Most productive authors and institutions

**Figure 5:**
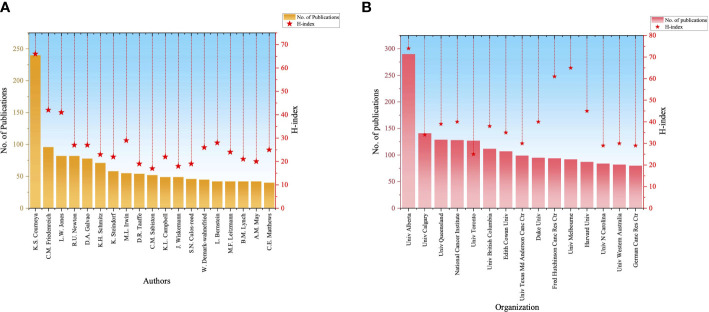
Distribution of the most influential authors and institutions. **(A)**The most productive authors. **(B)** The most productive institutions.

The most influential authors identified, based on the number of publications ≥ 40, are listed in **Figure 5A**; These prominent contributors are mainly from Canada, the United States, and Australia. The most prolific author was K.S. Courneya, with 240 publications during the last 20 years, who also received the highest number of citations (7,719) and H-index (66). C.M. Friedenreich and L.W. Jones ranked second and third, respectively. In addition, we analyzed the collaborations of highly productive authors in the field (**Supplementary Figure 1**) and identified several key research teams, including K.S. Courneya, R.U. Newton, and K.H. Schmitz. These authors are leading independent research teams that each have strong connections with various other authors. Further, we analyzed the most productive institutions (**Figure 5B**), based on the number of publications ≥ 80, and found that the University of Alberta, the University of Calgary, and the University of Queensland were the top three institutions, with the highest numbers of published papers, with the University of Alberta had the highest H-index. Details of the top 30 most productive authors and institutions are provided in **Supplementary Tables 2**, **3**.

### 3.5 Analysis of key publications

Reference analysis is an important strategy for assessing the impact of individual publications/research. In addition, mining and analysis of highly cited studies can help identify research hotspots and inform subsequent research. Here, we reported the top 10 publications with the minimum number of total citations over 600 (29–38). Holmes et al. conducted a prospective observational study to assess the effect of PA on the risk of death from breast cancer, and concluded that survivors of breast cancer who walked an average of 3–5 hours per week had the lowest risk of death (29). In addition, PA had a significant positive effect on self-esteem and chemotherapy completion rates of patients with cancer (34). Several studies have synthesized the potential associations of PA and SB in patients with cancer; in general, they found that appropriate PA is beneficial for both tumorigenic burden and prognosis of cancer survivors (30, 32, 33, 35, 38). Notably, a study by Moore et al. found that PA was associated with a high risk of malignant melanoma and prostate cancer (35). Rock et al. provided guidelines and recommendations of PA for patients with cancer (at least 150 min of exercise per week) (31). Given the numerous outstanding publications in the field, **Supplementary Table 4** shows the top 100 most cited publications.

### 3.6 Analysis of hotspots and trends in research

**Figure 6:**
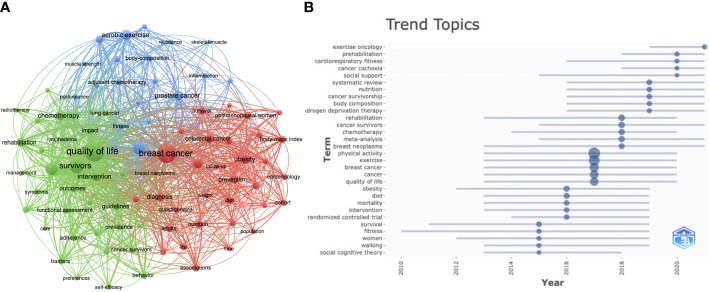
Analysis of Hotspots and Trends in Research. **(A)** Keyword co-occurrence visualization map. **(B)**Timeline of research trends in the field of PA, SB, and cancer.

VOSviewer was used to analyze and visualize ‘Keywords’ and ‘Keywords Plus’ in publications, to identify research hotspots and future frontiers. **Figure 6A** shows high frequency (> 80) keyword co-occurrence mapping in the field of PA and SB in cancer. We modified some keywords; for example, we unified “health-related quality of life” and “quality-of-life” as “quality of life”. We also removed some descriptive keywords such as “physical activity”, “sedentary behavior”, “exercise”, “health”, and “cancer”. The remaining core keywords can be divided into three clusters: (i) “breast cancer”, (ii) “quality of life”, and (iii) “aerobic exercise”. After that, we ranked the time of occurrence of all keywords to identify potential research trends and future frontiers. **Figure 6B** shows that research trends in the past 5 years have included exercise oncology, remote intervention, and social support for PA, among others. In addition, the research hotpots and trends topics on PA and SB in the context of cancer were summarized based on the PICOS statement (**Supplementary Table 5**).

## 4 Discussion

### 4.1 Summary of major findings

Here, we conducted a bibliometric analysis of publications on PA and SB in the context of cancer over the last two decades. Overall, we found that a growing number of studies have focused on potential correlations between exercise and cancer. Further, we found a significant increase in the number of publications and citations in this field from 2001 onwards, for two main reasons: first, unhealthy behaviors are becoming more prevalent in the population, leading to a significant increase in potential risk factors for cancer compared to two decades ago (5); and second, patients are surviving for longer periods, benefiting from improvements in medical care, therefore, how to achieve a better QoL for cancer survivors has become a hot topic (39, 40). In addition, the potential role of exercise in the primary prevention of cancer has received increasing attention, both from researchers and policymakers (41).We found that the United States was the most influential country, followed by Canada, Australia, the United Kingdom, China, and Germany, with researchers from these countries contributing 90% of publications. The United States dominates in terms of publication numbers and citations, making prominent contributions to advancement of research into PA and SB in cancer. The prominent contribution from the USA is due to its greater number of researchers and world-leading research institutions. In addition, the US investment in research funding has led to greater research opportunities and wider international collaborations. Cancer remains a global challenge, which requires closer cooperation among countries to overcome bottlenecks. In terms of international cooperation, the United States, Canada, and the United Kingdom cooperate more closely with other countries, which is a potential reason for their high citation rates. The University of Alberta, Canada, has the highest number of publications and H-Index, indicating that the institution publishes higher-quality articles and could be considered for further collaboration and study. In terms of authors, K.S. Courneya, C.M. Friedenreich, and L. W. Jones had the highest H-index values, indicating that they published higher-quality studies. We found that these authors have much larger publications and early pioneers in the field (10 years or more). They have published more articles in top journals and have therefore received more citations. In addition, it is easy to see that most of these authors are from the world’s leading research institutions and may have an excellent research team with more grad students and researchers to support their more in-depth studies. In addition to publishing many high-quality controlled clinical studies, their published comprehensive reviews in the field have been widely recognized. The journals that have published the most articles on PA and SB in cancer were *Supportive Care in Cancer*, *Cancer Epidemiology Biomarkers & Prevention*, and *BMC Cancer*. Most publications in this field focus on supportive care issues in cancer, especially symptoms and QoL, which may align with these journals’ scope. In addition, most studies are controlled clinical trials with small sample sizes, which may be easier to accept in comprehensive journals rather than top journals. We found that randomized controlled trials (RCTs) with large samples were more likely to be published in top journals and to receive more citations.

### 4.2 Research hotspots and trends

Based on the analysis of keyword co-occurrence and review of the timelines of research topic publication, we classified research hotspots and trends in the field of PA and SB in cancer into the topics summarized in the following sections.

#### 4.2.1 SB and PA in patients with breast cancer

Breast cancer is the most common cancer in women, with more than 10% of new cases per year (1). Previous studies have reported that SB accounts for 70% of modifiable breast cancer risk factors (42). The standardized incidence of breast cancer is significantly higher in sedentary women than in those who sit for less time (43). In addition, early diagnosis and increased awareness have improved overall survival, with the five-year survival for breast cancer survivors in the United States to be 90% in 2022 (44). As a result, more researchers are focusing on symptom management, prognosis, and QoL in female survivors. They have been granted more special funding opportunities focused on breast cancer. SB and low PA levels are common in survivors of breast cancer. Phillips et al. showed that the duration (555.7 min) of SB and proportion (66.4%) of survivors of breast cancer performing SB activities were 55 min and 7% higher, respectively, than those in healthy control populations (45). Only 11% of patients had a PA level that met the guideline-recommended criteria (cumulative 95 min of moderate-to-vigorous PA level) (46). Notably, although the current meta-analysis found that SB increased the risk of breast cancer by 8-17%, the conclusions were not entirely consistent (47). The 2018 Expert Report of the World Cancer Research Fund/American Institute for Cancer Research indicated that the current strength of evidence relating SB to breast cancer risk are limited (48). More evidence support from high-quality RCTs is needed, which is why researchers continually focus on this research hotspot. Finally, the biological mechanisms between PA and breast cancer are not yet clear and may involve adipokines and estrogen levels, inflammation, and oxidative stress (49), which have attracted a large number of preclinical studies.

#### 4.2.2 QoL issues in cancer survivors

Health-related quality of survival (HRQoL) has become an important factor in assessing clinical efficacy (50). In general, survivors of cancer have greater mental health needs, higher levels of anxiety and depression, and poorer HRQoL related to physical and mental health (51). Due to the immediate and long-term effects of cancer and its treatment, cancer survivors may suffer physical and psychological distress, including fatigue, decreased physical performance, depression, and anxiety (52, 53). QoL is a key advantage of PA, and many studies have confirmed that PA enhances QoL (54, 55). Cancer survivors who participated in PA activities had significantly higher QoL than those who did not (56). However, more detail should be provided on these associations. Several studies have identified ethnic/racial differences in PA levels in breast and colon cancer patients (57, 58). More research is still needed to reveal PA differences in less common cancers for population subgroups. Moreover, future studies should consider the association between PA and other cancer outcomes, including symptoms, treatment side effects, and prognosis, to determine dose-response relationships between PA and QoL, and to establish mechanisms to explain these associations. Finally, the emotion domains are key components of QoL. Clinical research should pay more attention to the psychiatric factors that influence PA in cancer survivors, including cancer-caused fatigue, anxiety, depression, self-efficacy, and health beliefs and engagement.

#### 4.2.3 Aerobic exercise

Lung cancer consistently ranks high among malignancies, in terms of annual incidence and mortality (44). Exercise training is safe, feasible, and effective in improving the prognosis of patients with lung cancer, particularly those with non-small cell lung cancer (59). Inspiratory muscle training and aerobic exercise improve lung function and respiratory muscle strength in postoperative patients with lung cancer, reducing the risk of sputum retention and postoperative pulmonary complications (60, 61). A meta-analysis showed that exercise training reduced overall and clinically relevant postoperative complications in patients with non-small cell lung cancer, compared with usual care (62). Aerobic exercise may improve sleep disturbance, psychological burden, and cancer treatment-induced cognitive impairment in survivors of breast and colon cancer (63–65). Notably, walking (aerobic) is the easiest and most preferred and accessible exercise for cancer survivors. There is still a need for research on other types and forms of exercise, such as high-intensity interval training, resistance training (RT), and comprehensive training. An integrated exercise protocol may be applicable to cancer survivors, which requires more exploratory studies. In summary, the development of future exercise programs requires the involvement of professionals and community support, including oncologists, physiotherapists, researchers, and patient associations (66).

#### 4.2.4 Cancer prehabilitation programs and cardiorespiratory fitness

Cancer prehabilitation programs have become a trending topic in the most recent timeline. In the current literature, a wide range of preoperative interventions are referred to as prehabilitation. Cancer patients undergoing surgery are at risk for delayed recovery. Prehabilitation aims to improve the patient’s preoperative function for better surgical tolerance and promote recovery (67). Most supporting evidence came from patients undergoing resection for colorectal, lung, and breast cancer (68–70). Previous studies have demonstrated the limited impact of exercise-only prehabilitation programs, while multimodal prehabilitation, including nutritional optimization, combined exercise programs (cardiorespiratory fitness and RT), and psychological well-being, have become the preferred option (71). This multimodal prehabilitation program seems to be further important in pancreatic cancer, where the diagnosis is often closely associated with cachexia and malnutrition (72). However, researchers have yet to be a consensus about what and when a prehabilitation program should take place. Therefore, more research should focus on the opportune time and exact protocols for preventative interventions. Considering the individual heterogeneity of patient physical and mental status, an individualized exercise rehabilitation protocol is essential to obtain long-term PA levels (73, 74).

#### 4.2.5 Remote intervention and social support for PA

PA of cancer survivors is a long-term, continual process. PA can reduce cancer side effects, but participation rates among cancer survivors are weak (13%–40%) (75). Telemedicine models, including mobile health (mHealth) and electronic health (eHealth), are emerging concepts in modern medical care that offer opportunities to improve PA for cancer survivors and reduce SB (76, 77). In the background of sudden public health events (e.g., the emergence of Covid-19 and Monkeypox virus), patients undergoing cancer treatment may be unable to take part in physical exercise. Many healthcare organizations have adopted telemedicine to avoid interruptions in treatment services, due to social distancing measures, isolation, self-isolation, and hospital visitor restrictions (78). Compared with the healthy population, patients with cancer may be more susceptible to novel coronavirus infections and serious complications (79, 80). Therefore, the use of telerehabilitation programs for patients with chronic diseases is highly recommended (81). A clear advantage of telemedicine is that cancer survivors can receive professional guidance from a physical therapist at home, thereby reducing the need for nonessential contact (82). Telemedicine can be divided into four main areas: web-based, telephone interventions, mobile applications, and SMS messaging. A systematic review that included 3,698 subjects revealed that participants showed good compliance and symptom relief from telemedicine (83); however, existing clinical trials have limitations, such as small sample size, non-randomized design, subject bias, single tumor type (mostly breast cancer survivors), and poor PA measurements (84), which may result in non-significant findings when more stringent inclusion criteria are used in meta-analysis (85). New methods are needed in the future to promote and support PA levels in cancer survivors.

## 5 Limitations

This study has some limitations. (i) Due to software limitations, we were unable to perform cross-check analysis of other high-quality databases (e.g., PubMed, Scopus). (ii) We only included studies in English, and some relevant articles in other languages may have been excluded. (iii) Only studies since 2001 were included, which may cause some omissions. (iv) In addition to the “keywords” provided by the author, we have included “keywords plus” to direct the searches, which may limit the results found. (v) Due to the existence of some synonymous keywords, bias may still exist, despite our efforts to standardize them.

## 6 Conclusions

This study analyzes publications related to PA and SB research in the context of cancer, and summarises the most influential authors, countries, institutions, and journals. In a word, evidence from the study results supports our hypothesis. Exercise oncology is a broad research topic focusing on cancer prevention, management, and supportive care. The current research focused on exercise and sedentariness in breast cancer patients and the role of PA in improving quality of life in survivorship. Emerging research foci were generally around cancer prehabilitation programs and remote intervention issues for PA. Furthermore, this analysis serves to support the prioritization of research topics in this field as it provides insight on gaps and deficiencies in the current literature. Future research should consider these shortcomings as a fundamental basis for study design.

## Data availability statement

The original contributions presented in the study are included in the article/**Supplementary Material**. Further inquiries can be directed to the corresponding authors.

## Author contributions

Conceptualization, JG. Methodology, MH. Software, YC. Data Curation, JY, YJ, and JH. Writing – Original Draft Preparation, JG. Writing – Review & Editing, GW. Supervision, JH. Funding Acquisition, JH and YJ. All authors contributed to the article and approved the submitted version.

## Supplementary material

Supplementary FIGURE 1 The collaborative relationships between different authors.
