# Supplementary figures and images for: Bibliometric analysis of global research on physical activity and sedentary behavior in the context of cancer
Source: Front Oncol. 2023 Jan 26;13:1095852. doi: 10.3389/fonc.2023.1095852 (PMC9909561; doi:10.3389/fonc.2023.1095852)

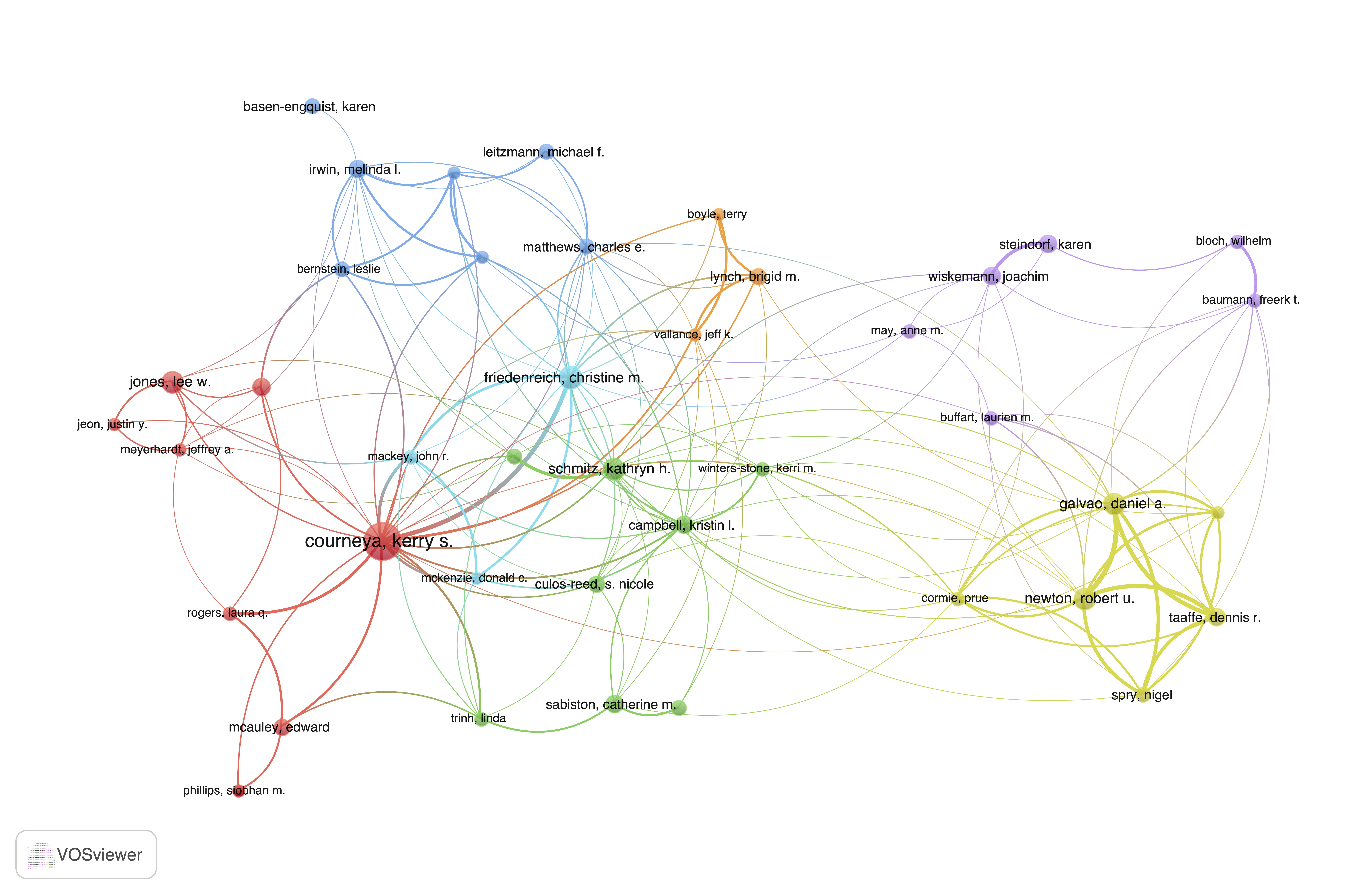

Supplement: Supplementary FIGURE 1 — The collaborative relationships between different authors. [file Image_1.jpeg]
