# Supplementary material for: Bibliometric analysis of global research on physical activity and sedentary behavior in the context of cancer
Source: Front Oncol. 2023 Jan 26;13:1095852. doi: 10.3389/fonc.2023.1095852 (PMC9909561; doi:10.3389/fonc.2023.1095852)
Supplement: Supplementary file 2 [file Table_1.docx]

Supplementary Table 1. Details of the search strategy.

| Number | Search term |
| --- | --- |
| 1 | TS=(physical activity) |
| 2 | TS=(motor activity) |
| 3 | TS=(outdoor activity) |
| 4 | TS=(exercise) |
| 5 | TS=(walk*) |
| 6 | TS=(active transport*) |
| 7 | TS=(sedentary) |
| 8 | TS=(sitting time) |
| 9 | TS=(active living) |
| 10 | TS=(leisure activity) |
| 11 | TS=(acceleromet*) |
| 12 | TS=(pedomet*) |
| 13 | TS=(cancer*) |
| 14 | TS=(tumor*) |
| 15 | TS=(tumour*) |
| 16 | 1 OR 2 OR 3 OR 4 OR 5 OR 6 OR 7 OR 8 OR 9 OR10 OR11 OR12 |
| 17 | 13 OR14 OR15 |
| 18 | 16 AND 17 |

TS = title, abstract, author keywords, and keywords plus.

* = any ending to the word.
