# Supplementary material for: Bibliometric analysis of global research on physical activity and sedentary behavior in the context of cancer
Source: Front Oncol. 2023 Jan 26;13:1095852. doi: 10.3389/fonc.2023.1095852 (PMC9909561; doi:10.3389/fonc.2023.1095852)
Supplement: Supplementary file 3 [file Table_2.docx]

Supplementary Table 2. The top 30 most productive authors in the field of physical activity and cancer between 2001 and 2022

| Rank | Author | Country | Article (n) | Total citations | Average citations | H-index |
| --- | --- | --- | --- | --- | --- | --- |
| #1 | K.S. Courneya | Canada | 240 | 7719 | 32.16 | 66 |
| #2 | C.M. Friedenreich | Canada | 96 | 2830 | 29.48 | 42 |
| #3 | L.W. Jones | USA | 82 | 2509 | 30.60 | 41 |
| #4 | R.U. Newton | Australia | 82 | 1364 | 16.63 | 27 |
| #5 | D.A. Galvao | Australia | 78 | 1418 | 18.18 | 27 |
| #6 | K.H. Schmitz | USA | 71 | 1502 | 21.15 | 23 |
| #7 | K. Steindorf | Germany | 58 | 838 | 14.45 | 22 |
| #8 | M.L. Irwin | USA | 55 | 1404 | 25.53 | 29 |
| #9 | D.R. Taaffe | Australia | 54 | 770 | 14.26 | 19 |
| #10 | C.M. Sabiston | Canada | 52 | 349 | - 1. 6.71 | 17 |
| #11 | K.L. Campbell | Canada | 49 | 946 | 19.31 | 22 |
| #12 | J. Wiskemann | UK | 49 | 567 | 11.57 | 18 |
| #13 | S.N. Culos-reed | Canada | 46 | 403 | 8.76 | 19 |
| #14 | W. Demark-wahnefried | USA | 45 | 956 | 21.24 | 26 |
| #15 | L. Bernstein | USA | 42 | 986 | 23.48 | 28 |
| #16 | M.F. Leitzmann | Germany | 42 | 626 | 14.90 | 24 |
| #17 | B.M. Lynch | Australia | 42 | 716 | 17.05 | 21 |
| #18 | A.M. May | Netherlands | 42 | 575 | 13.69 | 20 |
| #19 | C.E. Matthews | USA | 40 | 884 | 22.10 | 25 |
| #20 | L.M. Buffart | Netherlands | 38 | 671 | 17.66 | 18 |
| #21 | K. Basen-engquist | USA | 37 | 297 | 8.03 | 13 |
| #22 | J.R. Mackey | Canada | 37 | 2081 | 56.24 | 27 |
| #23 | F.T. Baumann | Germany | 36 | 387 | 10.75 | 17 |
| #24 | E. Mcauley | USA | 36 | 488 | 13.56 | 18 |
| #25 | N. Spry | Australia | 35 | 545 | 15.57 | 17 |
| #26 | J. Brunet | Canada | 34 | 267 | 7.85 | 14 |
| #27 | J.K. Vallance | Canada | 34 | 662 | 19.47 | 20 |
| #28 | J.C. Brown | USA | 32 | 338 | 10.56 | 18 |
| #29 | L. Trinh | Canada | 32 | 313 | 9.78 | 13 |
| #30 | D.C. Mckenzie | Canada | 31 | 980 | 31.61 | 21 |
