# Supplementary material for: Bibliometric analysis of global research on physical activity and sedentary behavior in the context of cancer
Source: Front Oncol. 2023 Jan 26;13:1095852. doi: 10.3389/fonc.2023.1095852 (PMC9909561; doi:10.3389/fonc.2023.1095852)
Supplement: Supplementary file 4 [file Table_3.docx]

Supplementary Table 3. The top 30 most productive institutions in the field of physical activity and cancer between 2001 and 2022

| Rank | Institutions | Country | No. of publications | Total citations | Average citations | H-index |
| --- | --- | --- | --- | --- | --- | --- |
| #1 | University of Alberta | Canada | 290 | 17875 | 61.64 | 74 |
| #2 | University of Calgary | Canada | 141 | 4097 | 29.06 | 34 |
| #3 | University of Queensland | Australia | 129 | 5673 | 43.98 | 39 |
| #4 | National Cancer Institute | USA | 128 | 8109 | 63.35 | 40 |
| #5 | University of Toronto | Canada | 127 | 1799 | 14.17 | 25 |
| #6 | University of British Columbia | Canada | 112 | 5835 | 52.10 | 38 |
| #7 | Edith Cowan University | Australia | 107 | 4100 | 38.32 | 35 |
| #8 | University of Texas MD Anderson Cancer Center | USA | 99 | 2405 | 24.29 | 30 |
| #9 | Duke University | USA | 95 | 4465 | 47.00 | 40 |
| #10 | Fred Hutchinson Cancer Research Center | USA | 94 | 6745 | 71.76 | 61 |
| #11 | University of Melbourne | Australia | 92 | 2443 | 26.55 | 65 |
| #12 | Harvard University | USA | 87 | 9355 | 107.53 | 45 |
| #13 | University of North Carolina | USA | 84 | 2281 | 27.15 | 29 |
| #14 | University of Western Australia | Australia | 82 | 2760 | 33.66 | 30 |
| #15 | German Cancer Research Center | Germany | 80 | 2815 | 35.19 | 29 |
| #16 | Dana-Farber Cancer Institute | USA | 74 | 5328 | 72.00 | 34 |
| #17 | Brigham and Women's Hospital | USA | 72 | 5074 | 70.47 | 40 |
| #18 | Memorial Sloan Kettering Cancer Center | USA | 70 | 2991 | 42.73 | 30 |
| #19 | University of Ottawa | Canada | 70 | 3353 | 47.90 | 29 |
| #20 | University of Alabama at Birmingham | USA | 67 | 3189 | 47.60 | 28 |
| #21 | University of Sydney | Australia | 65 | 3357 | 51.65 | 28 |
| #22 | University of Copenhagen | Denmark | 64 | 1986 | 31.03 | 25 |
| #23 | University of Pennsylvania | USA | 63 | 3401 | 53.98 | 27 |
| #24 | Queensland University of Technology | Australia | 62 | 1812 | 29.23 | 20 |
| #25 | Yale University | USA | 62 | 3514 | 56.68 | 31 |
| #26 | Karolinska Institute | Sweden | 61 | 2358 | 38.66 | 28 |
| #27 | University of Illinois | USA | 61 | 1994 | 32.69 | 27 |
| #28 | American Cancer Society | USA | 55 | 4586 | 83.38 | 28 |
| #29 | Harvard Medical School | USA | 55 | 2435 | 44.27 | 20 |
| #30 | University of Washington | USA | 55 | 3921 | 71.29 | 26 |
