# Supplementary material for: Bibliometric analysis of global research on physical activity and sedentary behavior in the context of cancer
Source: Front Oncol. 2023 Jan 26;13:1095852. doi: 10.3389/fonc.2023.1095852 (PMC9909561; doi:10.3389/fonc.2023.1095852)
Supplement: Supplementary file 5 [file Table_4.docx]

Supplementary Table 4. The Top 100 most cited publications in the field of physical activity and cancer between 2001 and 2022

| Rank | First author (Year) | Title | No. of citation | Journal (2021 IF) | DOI | Study design |
| --- | --- | --- | --- | --- | --- | --- |
| #1 | M.D. Holmes (2005) | Physical activity and survival after breast cancer diagnosis | 1196 | JAMA (157.36) | 10.1001/jama.293.20.2479 | Observational study |
| #2 | P.T. Katzmarzyk (2009) | Sitting time and mortality from all causes, cardiovascular disease, and cancer | 1106 | Med Sci Sports Exerc (6.29) | 10.1249/MSS.0b013e3181930355 | Observational study |
| #3 | C.L. Rock (2012) | Nutrition and physical activity guidelines for cancer survivors | 896 | CA Cancer J Clin (286.13) | 10.3322/caac.21142 | Review |
| #4 | R.M. Speck (2010) | An update of controlled physical activity trials in cancer survivors: a systematic review and meta-analysis | 856 | J Cancer Surviv (4.06) | 10.1007/s11764-009-0110-5 | SRMA |
| #5 | M.L. McNeely (2006) | Effects of exercise on breast cancer patients and survivors: a systematic review and meta-analysis | 713 | CMAJ (16.86) | 10.1503/cmaj.051073 | SRMA |
| #6 | K.S. Courneya (2007) | Effects of aerobic and resistance exercise in breast cancer patients receiving adjuvant chemotherapy: a multicenter randomized controlled trial | 703 | J Clin Oncol (50.72) | 10.1200/JCO.2006.08.2024 | RCT |
| #7 | S.C. Moore (2016) | Association of leisure-time physical activity with risk of 26 types of cancer in 1.44 million adults | 695 | JAMA Intern Med (44.41) | 10.1001/jamainternmed.2016.1548 | Observational study |
| #8 | N.J. Wareham (2003) | Validity and repeatability of a simple index derived from the short physical activity questionnaire used in the european prospective investigation into cancer and nutrition (epic) study | 639 | Public Health Nutr (4.54) | 10.1079/PHN2002439 | Methods |
| #9 | H.H. Kyu (2016) | Physical activity and risk of breast cancer, colon cancer, diabetes, ischemic heart disease, and ischemic stroke events: systematic review and dose-response meta-analysis for the global burden of disease study 2013 | 615 | BMJ (93.33) | 10.1136/bmj.i3857 | SRMA |
| #10 | J.A. Meyerhardt (2006) | Physical activity and survival after colorectal cancer diagnosis | 601 | J Clin Oncol (50.72) | 10.1200/JCO.2006.06.0855 | Observational study |
| #11 | R. Ballard-Barbash (2012) | Physical activity, biomarkers, and disease outcomes in cancer survivors: a systematic review | 568 | J Natl Cancer Inst (11.82) | 10.1093/jnci/djs207 | SRMA |
| #12 | S.I. Mishra (2012) | Exercise interventions on health-related quality of life for people with cancer during active treatment | 556 | Cochrane Database Syst Rev (12.01) | 10.1002/14651858.CD008465.pub2 | SRMA |
| #13 | R.J. Segal (2003) | Resistance exercise in men receiving androgen deprivation therapy for prostate cancer | 547 | J Clin Oncol (50.72) | 10.1200/JCO.2003.09.534 | RCT |
| #14 | K.S. Courneya (2003) | Randomized controlled trial of exercise training in postmenopausal breast cancer survivors: cardiopulmonary and quality of life outcomes | 537 | J Clin Oncol (50.72) | 10.1200/JCO.2003.04.093 | RCT |
| #15 | R. Patterson (2018) | Sedentary behaviour and risk of all-cause, cardiovascular and cancer mortality, and incident type 2 diabetes: a systematic review and dose response meta-analysis | 507 | Eur J Epidemiol (12.43) | 10.1007/s10654-018-0380-1 | SRMA |
| #16 | J.A. Meyerhardt (2006) | Impact of physical activity on cancer recurrence and survival in patients with stage iii colon cancer: findings from calgb 89803 | 507 | J Clin Oncol (50.72) | 10.1200/JCO.2006.06.0863 | Observational study |
| #17 | K.H. Schmitz (2005) | Controlled physical activity trials in cancer survivors: a systematic review and meta-analysis | 467 | Cancer Epidemiol Biomarkers Prev (4.09) | 10.1158/1055-9965.EPI-04-0703 | SRMA |
| #18 | A. McTiernan (2008) | Mechanisms linking physical activity with cancer | 456 | Nat Rev Cancer (69.80) | 10.1038/nrc2325 | Review |
| #19 | C.L. Rock (2012) | Nutrition and physical activity guidelines for cancer survivors | 453 | CA Cancer J Clin (286.13) | 10.3322/caac.21142 | Guideline |
| #20 | D.S. Michaud (2001) | Physical activity, obesity, height, and the risk of pancreatic cancer | 446 | JAMA (157.36) | 10.1001/jama.286.8.921 | Observational study |
| #21 | F. Cramp (2008) | Exercise for the management of cancer-related fatigue in adults | 429 | Cochrane Database Syst Rev (12.01) | 10.1002/14651858.CD006145.pub2 | SRMA |
| #22 | L. Pedersen (2016) | Voluntary running suppresses tumor growth through epinephrine- and il-6-dependent nk cell mobilization and redistribution | 425 | Cell Metab (31.37) | 10.1016/j.cmet.2016.01.011 | Preclinical study |
| #23 | R. Knols (2005) | Physical exercise in cancer patients during and after medical treatment: a systematic review of randomized and controlled clinical trials | 416 | J Clin Oncol (50.72) | 10.1200/JCO.2005.02.148 | SRMA |
| #24 | A.V. Patel (2010) | Leisure time spent sitting in relation to total mortality in a prospective cohort of us adults | 409 | Am J Epidemiol (5.36) | 10.1093/aje/kwq155 | Observational study |
| #25 | A. McTiernan (2003) | Recreational physical activity and the risk of breast cancer in postmenopausal women - the women's health initiative cohort study | 406 | JAMA (157.36) | 10.1001/jama.290.10.1331 | Observational study |
| #26 | R. Segal (2001) | Structured exercise improves physical functioning in women with stages i and ii breast cancer: results of a randomized controlled trial | 406 | J Clin Oncol (50.72) | 10.1200/JCO.2001.19.3.657 | RCT |
| #27 | S.I. Mishra (2012) | Exercise interventions on health-related quality of life for cancer survivors | 401 | Cochrane Database Syst Rev (12.01) | 10.1002/14651858.CD007566.pub2 | SRMA |
| #28 | E.M. Ibrahim (2011) | Physical activity and survival after breast cancer diagnosis: meta-analysis of published studies | 388 | Med Oncol (3.74) | 10.1007/s12032-010-9536-x | SRMA |
| #29 | R.J. Segal (2009) | Randomized controlled trial of resistance or aerobic exercise in men receiving radiation therapy for prostate cancer | 387 | J Clin Oncol (50.72) | 10.1200/JCO.2007.15.4963 | RCT |
| #30 | S.A. Kenfield (2011) | Physical activity and survival after prostate cancer diagnosis in the health professionals follow-up study | 383 | J Clin Oncol (50.72) | 10.1200/JCO.2010.31.5226 | Observational study |
| #31 | K.M. Mustian (2017) | Comparison of pharmaceutical, psychological, and exercise treatments for cancer-related fatigue a meta-analysis | 378 | JAMA Oncol (33.01) | 10.1001/jamaoncol.2016.6914 | SRMA |
| #32 | K.Y. Wolin (2009) | Physical activity and colon cancer prevention: a meta-analysis | 376 | Br J Cancer (9.08) | 10.1038/sj.bjc.6604917 | SRMA |
| #33 | D.Y. Fong (2012) | Physical activity for cancer survivors: meta-analysis of randomised controlled trials | 374 | BMJ (93.33) | 10.1136/bmj.e70 | SRMA |
| #34 | D.A. Galvao (2005) | Review of exercise intervention studies in cancer patients | 373 | J Clin Oncol (50.72) | 10.1200/JCO.2005.06.085 | SRMA |
| #35 | M.L. Irwin (2008) | Influence of pre- and postdiagnosis physical activity on mortality in breast cancer survivors: the health, eating, activity, and lifestyle study | 361 | J Clin Oncol (50.72) | 10.1200/JCO.2007.15.9822 | Observational study |
| #36 | E.M. Monninkhof (2007) | Physical activity and breast cancer - a systematic review | 360 | Epidemiology (4.86) | 10.1097/01.ede.0000251167.75581.98 | SRMA |
| #37 | C.N. Holick (2008) | Physical activity and survival after diagnosis of invasive breast cancer | 352 | ancer Epidemiol Biomarkers Prev (4.09) | 10.1158/1055-9965.EPI-07-0771 | Observational study |
| #38 | D. Schmid (2014) | Association between physical activity and mortality among breast cancer and colorectal cancer survivors: a systematic review and meta-analysis | 345 | Ann Oncol (51.77) | 10.1093/annonc/mdu012 | SRMA |
| #39 | V. Mock (2001) | Fatigue and quality of life outcomes of exercise during cancer treatment. | 332 | Cancer Pract | 10.1046/j.1523-5394.2001.009003119.x | Observational study |
| #40 | C.M. Friedenreich (2010) | State of the epidemiological evidence on physical activity and cancer prevention | 310 | Eur J Cancer (10) | 10.1016/j.ejca.2010.07.028 | Review |
| #41 | L. Adamsen (2009) | Effect of a multimodal high intensity exercise intervention in cancer patients undergoing chemotherapy: randomised controlled trial | 304 | BMJ (93.33) | 10.1136/bmj.b3410 | RCT |
| #42 | J. Kerr (2017) | Physical activity, sedentary behaviour, diet, and cancer: an update and emerging new evidence | 300 | Lancet Oncol (54.43) | 10.1016/S1470-2045(17)30411-4 | Review |
| #43 | P. Cormie (2017) | The impact of exercise on cancer mortality, recurrence, and treatment-related adverse effects | 300 | Epidemiol Rev (4.28) | 10.1093/epirev/mxx007 | SRMA |
| #44 | A.W. Moses (2004) | Reduced total energy expenditure and physical activity in cachectic patients with pancreatic cancer can be modulated by an energy and protein dense oral supplement enriched with n-3 fatty acids | 294 | Br J Cancer (9.08) | 10.1038/sj.bjc.6601620 | Observational study |
| #45 | K.S. Courneya (2003) | Exercise in cancer survivors: an overview of research | 290 | Med Sci Sports Exerc (6.29) | 10.1249/01.MSS.0000093622.41587.B6 | SRMA |
| #46 | S.F. Duijts (2011) | Effectiveness of behavioral techniques and physical exercise on psychosocial functioning and health-related quality of life in breast cancer patients and survivors-a meta-analysis | 289 | Psychooncology (3.96) | 10.1002/pon.1728 | SRMA |
| #47 | I.M. Lahart (2015) | Physical activity, risk of death and recurrence in breast cancer survivors: a systematic review and meta-analysis of epidemiological studies | 284 | Acta Oncol (4.31) | 10.3109/0284186X.2014.998275 | SRMA |
| #48 | L.M. Buffart (2017) | Effects and moderators of exercise on quality of life and physical function in patients with cancer: an individual patient data meta-analysis of 34 rcts | 277 | Cancer Treat Rev (13.61) | 10.1016/j.ctrv.2016.11.010 | SRMA |
| #49 | N. Mutrie (2007) | Benefits of supervised group exercise programme for women being treated for early stage breast cancer: pragmatic randomised controlled trial | 276 | BMJ (93.33) | 10.1136/bmj.39094.648553.AE | RCT |
| #50 | K.H. Schmitz (2019) | Exercise is medicine in oncology: engaging clinicians to help patients move through cancer | 275 | CA Cancer J Clin (286.13) | 10.3322/caac.21579 | Review |
| #51 | I.M. Lee (2003) | Physical activity and cancer prevention - data from epidemiologic studies | 272 | Med Sci Sports Exerc (6.29) | 10.1249/01.MSS.0000093620.27893.23 | Review |
| #52 | K.S. Courneya (2003) | A randomized trial of exercise and quality of life in colorectal cancer survivors | 266 | Eur J Cancer Care (2.33) | 10.1046/j.1365-2354.2003.00437.x | RCT |
| #53 | C.M. Friedenreich (2001) | Physical activity and cancer prevention: from observational to intervention research | 264 | Cancer Epidemiol Biomarkers Prev (4.09) |  | Review |
| #54 | J.C. Brown (2011) | Efficacy of exercise interventions in modulating cancer-related fatigue among adult cancer survivors: a meta-analysis | 263 | Cancer Epidemiol Biomarkers Prev (4.09) | 10.1158/1055-9965.EPI-10-0988 | SRMA |
| #55 | A.M. Haydon (2006) | Effect of physical activity and body size on survival after diagnosis with colorectal cancer | 258 | Gut (31.79) | 10.1136/gut.2005.068189 | Observational study |
| #56 | C.E. Matthews (2007) | Influence of exercise, walking, cycling, and overall nonexercise physical activity on mortality in chinese women | 253 | Am J Epidemiol (5.36) | 10.1093/aje/kwm088 | Observational study |
| #57 | V. Mock (2005) | Exercise manages fatigue during breast cancer treatment: a randomized controlled trial | 253 | Psychooncology (3.96) | 10.1002/pon.863 | RCT |
| #58 | Y. Wu (2013) | Physical activity and risk of breast cancer: a meta-analysis of prospective studies | 249 | Breast Cancer Res Treat (4.62) | 10.1007/s10549-012-2396-7 | SRMA |
| #59 | B.M. Lynch (2010) | Sedentary behavior and cancer: a systematic review of the literature and proposed biological mechanisms | 249 | Cancer Epidemiol Biomarkers Prev (4.09) | 10.1158/1055-9965.EPI-10-0815 | SRMA |
| #60 | R.E. Patterson (2003) | Changes in diet, physical activity, and supplement use among adults diagnosed with cancer | 248 | J Am Diet Assoc | 10.1053/jada.2003.50045 | Observational study |
| #61 | W. Kuijpers (2013) | A systematic review of web-based interventions for patient empowerment and physical activity in chronic diseases: relevance for cancer survivors | 247 | J Med Internet Res (7.08) | 10.2196/jmir.2281 | Review |
| #62 | M.L. Irwin (2004) | Physical activity levels among breast cancer survivors | 246 | Med Sci Sports Exerc (6.29) | 10.1249/01.MSS.0000074670.03001.98 | Observational study |
| #63 | A.V. Patel (2019) | American college of sports medicine roundtable report on physical activity, sedentary behavior, and cancer prevention and control | 241 | Med Sci Sports Exerc (6.29) | 10.1249/MSS.0000000000002117 | Guideline |
| #64 | K.S. Courneya (2009) | Randomized controlled trial of the effects of aerobic exercise on physical functioning and quality of life in lymphoma patients | 239 | J Clin Oncol (50.72) | 10.1200/JCO.2008.20.0634 | RCT |
| #65 | J.K. Vallance (2007) | Randomized controlled trial of the effects of print materials and step pedometers on physical activity and quality of life in breast cancer survivors | 237 | J Clin Oncol (50.72) | 10.1200/JCO.2006.07.9988 | RCT |
| #66 | D. Romaguera (2012) | Is concordance with world cancer research fund/american institute for cancer research guidelines for cancer prevention related to subsequent risk of cancer? Results from the epic study | 236 | Z Am J Clin Nutr (8.47) | 10.3945/ajcn.111.031674 | Observational study |
| #67 | A.K. Samad (2005) | A meta-analysis of the association of physical activity with reduced risk of colorectal cancer | 233 | Samad AK (3.92) | 10.1111/j.1463-1318.2005.00747.x | Meta-analysis |
| #68 | A.McTiernan(2019) | Physical activity in cancer prevention and survival: a systematic review | 232 | Med Sci Sports Exerc (6.29) | 10.1249/MSS.0000000000001937 | SRMA |
| #69 | C.M. Friedenreich (2008) | Physical activity and breast cancer risk: impact of timing, type and dose of activity and population subgroup effects | 232 | Br J Sports Med (18.47) | 10.1136/bjsm.2006.029132 | SRMA |
| #70 | F.C. Dimeo (2001) | Effects of exercise on cancer-related fatigue | 223 | Cancer (6.92) | 10.1002/1097-0142(20010915)92:6+<1689::AID-CNCR1498>3.0.CO;2-H | Review |
| #71 | M.A. West (2015) | Effect of prehabilitation on objectively measured physical fitness after neoadjuvant treatment in preoperative rectal cancer patients: a blinded interventional pilot study | 222 | Br J Anaesth (11.72) | 10.1093/bja/aeu318 | Observational study |
| #72 | J.C. Brown (2012) | The efficacy of exercise in reducing depressive symptoms among cancer survivors: a meta-analysis | 222 | PLoS One (3.75) | 10.1371/journal.pone.0030955 | SRMA |
| #73 | T.I. Nilsen (2001) | Prospective study of colorectal cancer risk and physical activity, diabetes, blood glucose and bmi: exploring the hyperinsulinaemia hypothesis | 222 | Br J Cancer (9.08) | 10.1054/bjoc.2000.1582 | Observational study |
| #74 | D.C. McKenzie (2003) | Effect of upper extremity exercise on secondary lymphedema in breast cancer patients: a pilot study | 220 | J Clin Oncol (50.72) | 10.1200/JCO.2003.04.069 | Observational study |
| #75 | G.Hu,(2005) | The effects of physical activity and body mass index on cardiovascular, cancer and all-cause mortality among 47,212 middle-aged finnish men and women | 216 | Int J Obes (5.55) | 10.1038/sj.ijo.0802870 | Observational study |
| #76 | M.L. Irwin (2011) | Physical activity and survival in postmenopausal women with breast cancer: results from the women's health initiative | 214 | Cancer Prev Res (3.3) | 10.1158/1940-6207.CAPR-10-0295 | Observational study |
| #77 | K.S. Courneya (2003) | The group psychotherapy and home-based physical exercise (group-hope) trial in cancer survivors: physical fitness and quality of life outcomes | 213 | Psychooncology (3.96) | 10.1002/pon.658 | RCT |
| #78 | J.C. Brown (2012) | Cancer, physical activity, and exercise | 210 | Compr Physiol (8.92) | 10.1002/cphy.c120005 | Review |
| #79 | A.J. Daley (2007) | Randomized trial of exercise therapy in women treated for breast cancer | 206 | J Clin Oncol (50.72) | 10.1200/JCO.2006.09.5083 | RCT |
| #80 | T. Boyle (2012) | Physical activity and risks of proximal and distal colon cancers: a systematic review and meta-analysis | 200 | J Natl Cancer Inst (11.82) | 10.1093/jnci/djs354 | SRMA |
| #81 | C.G. Valle (2013) | A randomized trial of a facebook-based physical activity intervention for young adult cancer survivors | 198 | J Cancer Surviv (4.06) | 10.1007/s11764-013-0279-5 | RCT |
| #82 | L.W. Jones (2009) | Exercise intolerance in cancer and the role of exercise therapy to reverse dysfunction | 197 | Lancet Oncol (54.43) | 10.1016/S1470-2045(09)70031-2 | Review |
| #83 | L.W. Jones (2002) | Exercise counseling and programming preferences of cancer survivors | 196 | Cancer Pract | 10.1046/j.1523-5394.2002.104003.x | Observational study |
| #84 | E.L. Richman (2011) | Physical activity after diagnosis and risk of prostate cancer progression: data from the cancer of the prostate strategic urologic research endeavor | 194 | Cancer Res (13.31) | 10.1158/0008-5472.CAN-10-3932 | Observational study |
| #85 | M.L. Irwin (2009) | Randomized controlled trial of aerobic exercise on insulin and insulin-like growth factors in breast cancer survivors: the yale exercise and survivorship study | 192 | Cancer Epidemiol Biomarkers Prev (4.09) | 10.1158/1055-9965.EPI-08-0531 | RCT |
| #86 | A.L. Schwartz (2001) | Exercise reduces daily fatigue in women with breast cancer receiving chemotherapy | 191 | Med Sci Sports Exerc (6.29) | 10.1097/00005768-200105000-00006 | Observational study |
| #87 | S.N. Culos-Reed (2006) | A pilot study of yoga for breast cancer survivors: physical and psychological benefits | 189 | Psychooncology (3.96) | 10.1002/pon.1021 | RCT |
| #88 | M.L. McCullough (2011) | Following cancer prevention guidelines reduces risk of cancer, cardiovascular disease, and all-cause mortality | 188 | Cancer Epidemiol Biomarkers Prev (4.09) | 10.1158/1055-9965.EPI-10-1173 | Observational study |
| #89 | J.A. Meyerhardt (2009) | Physical activity and male colorectal cancer survival | 188 | Arch Intern Med | 10.1001/archinternmed.2009.412 | Observational study |
| #90 | F.G. Stacey (2015) | A systematic review and meta-analysis of social cognitive theory-based physical activity and/or nutrition behavior change interventions for cancer survivors | 187 | J Cancer Surviv (4.06) | 10.1007/s11764-014-0413-z | SRMA |
| #91 | N.J. Davies (2011) | The role of diet and physical activity in breast, colorectal, and prostate cancer survivorship: a review of the literature | 187 | Br J Cancer (9.08) | 10.1038/bjc.2011.423 | SRMA |
| #92 | A.V. Patel (2005) | Obesity, recreational physical activity, and risk of pancreatic cancer in a large us cohort | 187 | Cancer Epidemiol Biomarkers Prev (4.09) | 10.1158/1055-9965.EPI-04-0583 | Observational study |
| #93 | L.M. Buffart (2012) | Physical and psychosocial benefits of yoga in cancer patients and survivors, a systematic review and meta-analysis of randomized controlled trials | 184 | BMC Cancer (4.64) | 10.1186/1471-2407-12-559 | SRMA |
| #94 | R.A. Ferrer (2011) | Exercise interventions for cancer survivors: a meta-analysis of quality of life outcomes | 184 | Ann Behav Med (4.87) | 10.1007/s12160-010-9225-1 | SRMA |
| #95 | A. Lucia (2003) | Cancer-related fatigue: can exercise physiology assist oncologists? | 183 | Lancet Oncol (54.43) | 10.1016/S1470-2045(03)01221-X | Review |
| #96 | D.W. Pekmezi (2011) | Updated evidence in support of diet and exercise interventions in cancer survivors | 182 | Acta Oncol (4.31) | 10.3109/0284186X.2010.529822 | SRMA |
| #97 | T.R. Burnham (2002) | Effects of exercise on physiological and psychological variables in cancer survivors | 182 | Med Sci Sports Exerc (6.29) | 10.1097/00005768-200212000-00001 | Observational study |
| #98 | K.S. Courneya (2013) | Effects of exercise dose and type during breast cancer chemotherapy: multicenter randomized trial | 180 | J Natl Cancer Inst (11.82) | 10.1093/jnci/djt297 | RCT |
| #99 | M.J. Velthuis (2010) | The effect of physical exercise on cancer-related fatigue during cancer treatment: a meta-analysis of randomised controlled trials | 180 | Clin Oncol (4.93) | 10.1016/j.clon.2009.12.005 | SRMA |
| #100 | J.K. Kiecolt-Glaser (2014) | Yoga's impact on inflammation, mood, and fatigue in breast cancer survivors: a randomized controlled trial | 178 | J Clin Oncol (50.72) | 10.1200/JCO.2013.51.8860 | RCT |

Abbreviations: SRMA, Systematic review and meta-analysis; RCT, Randomized controlled trials
