# Supplementary material for: Bibliometric analysis of global research on physical activity and sedentary behavior in the context of cancer
Source: Front Oncol. 2023 Jan 26;13:1095852. doi: 10.3389/fonc.2023.1095852 (PMC9909561; doi:10.3389/fonc.2023.1095852)
Supplement: Supplementary file 6 [file Table_5.docx]

Supplementary Table 5. The PICOS format of hot topics in the field of physical activity and cancer between 2001 and 2022

| Parameter | Key words |
| --- | --- |
| P: Population | Prehabilitation, Androgen deprivation therapy, rehabilitation, cancer survivors, chemotherapy, radiotherapy, breast, lung, colorectal, diet, mortality, women, nutrition |
| I: Intervention | Exercise, walking, aerobic, social support, social cognitive theory |
| C: Comparison | NA |
| O: Outcome | Cachexia, fitness, QoL, body composition, muscle strength |
| S: Study design | Systematic review, meta-analysis, RCT, guidelines |

Abbreviation: NA, not applicable; QoL, quality of life; RCT, Randomized controlled trial
